# Why repair fails in AQP4-IgG-positive neuromyelitis optica spectrum disease: an astrocyte-centered framework for remyelination and neuroaxonal preservation

**DOI:** 10.3389/fimmu.2026.1889927

**Published:** 2026-09-29

**Authors:** Wenjing Luo, Ni Peng, Siqian Wei, Shuxin Huang, Yixin Miao, Yi Du

**Affiliations:** 1 Department of Neurology, The First Affiliated Hospital of Guangxi Medical University, Nanning, China; 2 Department of Ophthalmology, The First Affiliated Hospital of Guangxi Medical University, Nanning, China; 3 Department of Ophthalmology, The Second Affiliated Hospital of Guangxi Medical University, Nanning, China

**Keywords:** Aquaporin-4 immunoglobulin G, astrocytopathy, microglia, neuroaxonal preservation, neuromyelitis optica spectrum disease, oligodendrocyte precursor cell, remyelination, repair competence

## Abstract

Aquaporin-4 immunoglobulin G (AQP4-IgG)-positive neuromyelitis optica spectrum disease (NMOSD) is an autoimmune astrocytopathy in which a single attack may cause permanent visual or neurological disability. Although targeted immunotherapies reduce relapse risk, they do not establish repair of previously injured tissue. Repair failure is framed as disruption of an astrocyte-organized environment required for glial communication, oligodendrocyte lineage progression, myelin formation, and axonal survival. Five linked barriers organize the review: loss of astrocytic homeostatic support, glial uncoupling, oligodendrocyte precursor cell maturation stall, depletion of repairable substrate, and failure to transition from injury resolution to active repair. Human pathology and experimental studies show that astrocyte survival, repopulation, or barrier resealing does not necessarily restore the functions required for remyelination. Microglia and macrophages, peripheral immune activity, organelle and metabolic stress, and the neurovascular and extracellular matrix environment further modify repair capacity. Delayed-treatment studies of clobetasol and edaravone provide the strongest disease-relevant preclinical evidence that myelin recovery can be enhanced after demyelination has developed, whereas most other strategies remain indirect or repair-permissive. These findings do not establish human efficacy; a human remyelination claim would require convergent evidence of new myelin, improved conduction, and durable regional benefit. Priorities include disease-specific delayed-treatment models, direct confirmation of newly formed myelin, and testing under persistent AQP4-IgG-related injury. As of 24 August 2026, no controlled human study has demonstrated remyelination of an established AQP4-IgG-positive NMOSD lesion.

## 1 Introduction

Aquaporin-4 immunoglobulin G (AQP4-IgG)-positive neuromyelitis optica spectrum disease (NMOSD) is a relapsing autoimmune inflammatory disease of the central nervous system (CNS) in which astrocytes are the primary tissue target (1, 2). The optic nerves and spinal cord bear much of the clinical burden, and a single severe attack can cause irreversible blindness, paralysis, pain or autonomic dysfunction. AQP4-IgG binding can activate complement-dependent cytotoxicity and antibody-dependent cellular cytotoxicity, perturb astrocytic glutamate transport and recruit granulocytes and other inflammatory effector cells. Oligodendrocyte injury, demyelination and axonal loss then emerge within an evolving multicellular lesion (1–3).

Controlled trials have established effective relapse prevention with B-cell lineage depletion, interleukin-6 receptor blockade and terminal complement inhibition (4–6). These therapies have changed prognosis, yet preventing another attack does not reconstruct tissue already damaged by a previous attack. Clinical improvement after acute treatment does not by itself establish remyelination: it may reflect reduced edema, reversal of conduction block, metabolic recovery in surviving fibers, compensation or rehabilitation.

Repair in AQP4-IgG-positive NMOSD cannot simply be imported from multiple sclerosis. Multiple sclerosis has generated a large remyelination literature, but the initiating lesion in AQP4-IgG-positive NMOSD is an immune attack on the cell that organizes extracellular ion and water balance, glutamate clearance, metabolic support, blood-brain barrier function and intercellular glial communication. The biological question therefore extends beyond whether oligodendrocyte precursor cells (OPCs) remain: has the lesion regained the conditions in which those cells can mature, form stable myelin and support surviving axons (7, 8)?

Remyelination may itself be neuroprotective because newly formed myelin can restore saltatory conduction and metabolic support to an intact axon. A Brain scientific commentary developed this argument and emphasized that the therapeutic window depends on age, time since injury and the degree of axonal abnormality (9). That argument is biologically relevant to NMOSD but remains indirect evidence. It was not derived from AQP4-IgG-mediated lesions and cannot establish that remyelination will rescue an already destructive NMOSD lesion.

Recent reviews have synthesized NMOSD immunopathogenesis, experimental models, and microglial biology (10, 11). Here, an astrocyte-centered framework is used to examine why AQP4-IgG-mediated lesions may fail to progress from injury resolution to effective remyelination and neuroaxonal preservation. Five linked barriers provide the top-level organization, while microglia and macrophages, peripheral immune activity, organelle stress, the neurovascular unit, and the extracellular matrix are treated as cross-cutting determinants that can modify several barriers. The review distinguishes direct AQP4-IgG-specific evidence from inference based on related demyelinating systems and identifies experiments that can separate tissue preservation, repair-permissive effects, and remyelination. Human implications are considered where they clarify how a preclinical repair signal might be evaluated.

## 2 Literature search and evidence boundaries

PubMed was searched without a date restriction and last updated on 24 August 2026. The search was supplemented by backward citation checking, targeted publisher-site title and digital object identifier searches for online-first records, and a ClinicalTrials.gov search. Search terms covered repair and recovery; cellular and lesion environment mechanisms; peripheral and acute immune effectors; regional measurement and biomarkers; candidate interventions; and targeted astrocyte, myeloid and organelle mechanisms. English-language records and records with an English abstract were considered.

Eligible evidence addressed AQP4-IgG-positive or serostatus-unspecified NMOSD repair biology, treated recovery, measurement or translation. Mixed optic neuritis cohorts were retained only when the disease mix was reported or the method informed regional measurement. Multiple sclerosis and generic demyelination studies were used to fill an explicit disease-specific gap, not as efficacy evidence for NMOSD. Commentaries were used only for interpretation. Contradictory findings and negative primary outcomes were retained when relevant. Registered studies were included to describe the translational pipeline, not as evidence of efficacy.

Because this was a narrative review, evidence was interpreted according to disease specificity and study design. Direct human observations and AQP4-IgG-relevant experimental studies were given greater weight than evidence extrapolated from multiple sclerosis or generic demyelination models. Experimental findings were not assumed to represent clinical repair, and relapse prevention, reduced inflammation, or cross-sectional imaging changes were not considered evidence of remyelination unless the underlying study directly supported that interpretation.

## 3 What constitutes repair in AQP4-IgG-positive NMOSD?

Repair terminology is often used imprecisely. **Table 1** summarizes the operational definitions used throughout this review. Disease control suppresses new inflammatory activity. Acute injury limitation reduces tissue damage during an attack. Substrate preservation retains the axons, glial lineage cells, vascular architecture and local tissue organization that subsequent repair would require. A repair-permissive intervention improves the lesion environment without necessarily reconstructing tissue. Neuroprotection limits injury to viable neurons or axons. Repair restores a structure–function relation after injury. Regeneration is a stronger claim that implies substantial axonal regrowth or replacement of lost cells.

**Table 1:** Operational distinctions used in this review.

Term	Operational definition	What the term does not prove	Typical evidence needed
Disease control	Suppression of new inflammatory activity, especially prevention of relapses or new lesions.	Repair of tissue injured during an earlier attack.	Relapse, new lesion or disease activity outcomes.
Acute injury limitation	Reduction of tissue damage during an active attack by interrupting antibody, complement, cellular or secondary injury.	Reconstruction of tissue already lost.	Early treatment comparison with acute injury and regional functional outcomes.
Substrate preservation	Retention of surviving axons, viable glial lineage cells, vascular integrity and local tissue organization needed for later repair.	Formation of new myelin or axonal regrowth.	Longitudinal structural and injury state measures.
Repair-permissive intervention	A treatment that restores conditions in which endogenous or treatment-induced repair can proceed.	Direct remyelination or reconstruction.	Prespecified environmental target engagement followed by an orthogonal downstream signal.
Neuroprotection	Limitation of neuronal or axonal injury during or shortly after an attack.	Reversal of established tissue loss.	Reduced injury biomarkers or structural loss with preserved regional function.
Biological repair	Intervention-linked restoration of a previously injured cellular or structural component.	Durable patient benefit in humans.	Prior injury, appropriate timing, cellular or structural reconstruction and separation from preservation alone.
Biological remyelination	New myelin formation around previously demyelinated surviving axons.	Replacement of lost axons, complete reversal of disability or clinical efficacy.	Lineage, pulse-labeling, ultrastructural or equivalent evidence tied to prior demyelination and preserved axons.
Clinically meaningful human repair	Durable restoration of a regional structure–function relation after injury.	Clinical stabilization, compensation or reduced inflammation alone.	Intervention-linked cellular or structural restoration, an orthogonal regional benefit, durability and accounting for ongoing injury and co-interventions.
Clinically meaningful human remyelination	Human repair with myelin-sensitive evidence compatible with new or restored myelin around preserved axons and improved conduction in the same pathway.	Axonal regeneration or full reversal of previous tissue loss.	Prespecified longitudinal convergence of myelin-sensitive, conduction and regional functional evidence.
Regeneration	Substantial reconstruction, such as axonal regrowth or replacement of lost cellular elements.	Partial physiological recovery in surviving pathways.	Direct evidence of regrowth or replacement with functional integration.

Definitions are operational and are not proposed as consensus criteria.

Biological remyelination should be distinguished from a clinically meaningful human remyelination claim. Biological remyelination denotes new myelin formation around previously demyelinated surviving axons. It can be demonstrated in an experimental system by appropriately timed lineage, ultrastructural or equivalent evidence even when a behavioral or electrophysiological signal is not detected. A clinically meaningful human claim requires more: evidence compatible with new or restored myelin around preserved axons, improved conduction in the same pathway, a durable regional benefit, and adequate accounting for continuing injury, spontaneous recovery and co-interventions.

These categories overlap in practice but should not be collapsed analytically. Rapid interruption of complement-mediated astrocyte injury may preserve oligodendrocytes and axons and shorten the period in which the lesion remains toxic. That can facilitate later recovery without being a direct remyelinating mechanism. Conversely, an agent that drives OPC differentiation may produce biological remyelination in a substrate-rich model yet fail clinically because too few axons survive, new sheaths are poorly integrated, exposure is inadequate, or the chosen outcome is insensitive.

Repair is also region-specific. In optic neuritis, a credible human claim may require concordance among optic nerve lesion context, preserved retinal ganglion cell axons, improved visual pathway conduction and durable visual function. In myelitis, the relevant structure–function relation is distributed across long spinal tracts and segmental circuits. Brainstem and area postrema lesions require another set of regional outcomes. A single global disability score cannot establish tissue repair in all three settings.

## 4 Why repair fails: lesion states and five linked barriers

### 4.1 Destructive, sublytic and repopulation-associated astrocyte states

A uniform “repair failure” model assumes that AQP4-IgG produces one lesion state. Human pathology argues against that view. Classic lesions show perivascular astrocyte destruction, loss of aquaporin-4 (AQP4) and glial fibrillary acidic protein (GFAP), complement deposition, inflammatory infiltration and secondary demyelination. Other specimens show reparative astrogliosis and newly generated perivascular astrocytes after acute destruction (12). An ordered histopathological classification places complement-active astrocyte injury, progenitor recruitment and later gliotic appearances at different putative stages (13). Because these specimens are cross-sectional, they cannot show that one lesion passed through those states in that order; destructive and reparative microdomains may coexist within the same lesion.

Sublytic astrocytopathy broadens the spectrum. In a study of 23 autopsy cases, reactive and dystrophic astrocyte changes extended beyond fully demyelinated lesions and could occur without complete AQP4 loss or terminal complement deposition. AQP4 transcripts were present in reactive astrocytes that lacked surface AQP4 protein (14). This supports dysregulated expression or localization; restoration is only one possible interpretation. Visible astrocytes can therefore remain functionally abnormal.

Recovery processes are also temporally dissociable. Blood–brain barrier resealing can occur independently of astrocyte regeneration, so disappearance of enhancement or gross barrier restoration does not show that astrocytic homeostasis has returned (15). In antibody-mediated lesions, astrocytes and oligodendroglial lineage cells can repopulate tissue while effective remyelination remains limited (16). Cell number, cell identity, functional state and successful integration are separate measurements.

Astrocyte regeneration is nevertheless biologically tractable. Fibroblast growth factor 8–developing brain homeobox 1 (FGF8–DBX1) signaling increased astrocyte regeneration after severe focal loss in rats and was accompanied by circuit reorganization and motor recovery (17). Longitudinal two-photon imaging after focal AQP4 antibody-mediated astrocyte ablation has now shown proliferation, prolonged multinucleated states, polarized process extension and gradual daughter nucleus translocation into previously depleted territories; comparable morphologies were observed in human AQP4-IgG-positive lesions (18). This study closes part of the temporal gap left by cross-sectional pathology, but the focal cortical model selectively depleted astrocytes in relatively preserved tissue and did not establish restoration of the full homeostatic program or remyelination.

These pathological states change the experimental target. A destructive lesion prioritizes immediate injury limitation and substrate preservation. A sublytic lesion may retain a window for restoring transport, metabolism, coupling or inflammatory control. A repopulated lesion raises a different question: whether returning cells have regained the functions that permit oligodendrocyte maturation and stable axonal support. None of these states is presently a validated bedside category.

### 4.2 Barrier 1: loss of astrocytic homeostatic support

Astrocytes regulate extracellular glutamate, potassium and water; provide metabolic and trophic support; participate in neurovascular regulation; and organize the local extracellular environment (19, 20). AQP4-IgG can downregulate excitatory amino acid transporter 2 (EAAT2) and impair glutamate handling in some experimental systems before complete astrocyte destruction (3). The extent and *in vivo* contribution are model-dependent, but the finding illustrates why GFAP staining alone does not define functional preservation.

Clinical experience that interferon beta is ineffective or may worsen NMOSD is a warning against importing multiple sclerosis treatment logic (21). In a 2026 experimental study, interferon beta ameliorated experimental autoimmune encephalomyelitis but worsened NMOSD-like astrocytopathy; deletion of type I interferon receptor 1 (IFNAR1) altered astrocyte activation and disease severity (22). This identifies astrocyte activation state as a mechanistic discriminator, not a simple “more versus less activation” therapeutic scale. Another AQP4-IgG-related study linked astrocytic signal transducer and activator of transcription 3–chitinase-3-like protein 1 (CHI3L1)–receptor for advanced glycation end products/nuclear factor kappa B (NF-κB) signaling to inflammatory gliosis, demyelination and motor deficits; pathway blockade reduced disease in the model (23). Because these interventions overlapped with evolving injury, reduced demyelination does not by itself prove post-injury repair.

The decisive experiment is functional rather than morphological. Astrocyte-directed benefit should be attributed only when a prespecified homeostatic or inflammatory function is restored, astrocyte-specific necessity or rescue is demonstrated, and downstream oligodendroglial, myelin and pathway outcomes improve. Restoring AQP4-expressing astrocytes also recreates the antibody target; persistent AQP4-IgG exposure or re-challenge therefore belongs in the safety design.

### 4.3 Barrier 2: glial uncoupling

Astrocytes and oligodendrocytes form connexin-based gap junction networks that exchange ions and small metabolites. NMOSD tissue shows connexin loss or mislocalization, while cultures treated with patient immunoglobulin G show reduced gap junction communication and increased connexin 43 hemichannel opening (24). These are related but distinct observations. Oligodendrocyte connexin 47 stability also depends on astrocytic connexin 43 expression in a non-NMOSD system (25).

The focal astrocyte ablation study adds an important qualification: astrocyte repopulation proceeded despite astrocyte-specific deletion of connexin 30 and connexin 43 (18). This does not show that glial coupling is irrelevant to remyelination. It shows that repopulation of astrocyte territory and restoration of an intercellular support network are separable. Future experiments should report connexin expression, channel localization, intercellular transfer and hemichannel activity as distinct endpoints, then link them to oligodendrocyte maturation, myelin structure and pathway physiology.

### 4.4 Barrier 3: oligodendrocyte lineage maturation stall

Remyelination requires more than the presence of OPCs. It requires recruitment, differentiation into mature oligodendrocytes, selection of viable axons, formation of compact myelin, restoration of nodal and paranodal organization, and sustained support of conduction. Antibody-mediated models of multiple sclerosis and NMOSD show distinct repair patterns, with less efficient remyelination in the NMOSD-like setting despite oligodendroglial repopulation (16).

The maturation checkpoint is pharmacologically tractable in disease-relevant models. Clobetasol promoted OPC differentiation and myelin recovery in organotypic and mouse models (26). In organotypic slices, clobetasol was introduced after 20 hours of AQP4-IgG and complement exposure and continued for 6 days after removal of the injurious stimulus. In a delayed-treatment mouse experiment, clobetasol was started 4 days after intracerebral AQP4-IgG and complement injection, when astrocyte injury and demyelination were already evident, and lesions were assessed on day 9. Clobetasol increased mature oligodendrocytes and myelin recovery without an apparent reduction in astrocyte injury or inflammatory staining, strengthening the interpretation that its effect was not explained solely by suppression of the initial inflammatory injury.

Edaravone likewise promoted OPC maturation and remyelination across ex vivo and *in vivo* models using patient-derived total immunoglobulin G from individuals with NMOSD (NMO-IgG) or monoclonal AQP4-IgG (27). In organotypic slices, edaravone was introduced after 48 hours of NMO-IgG and complement exposure and continued for 6 days after removal of the injurious stimulus. In a separate delayed-treatment experiment, edaravone was initiated 4 days after intracerebral NMO-IgG and human complement injection, when astrocyte injury and demyelination were already established, and greater myelin recovery was observed by day 10. Other experiments provided complementary evidence: ultrastructural analysis in a focal AQP4-IgG model showed more myelinated axons and a lower g-ratio than in vehicle-treated AQP4-IgG lesions, while a separate systemic NMO-IgG model showed improved motor performance. Pharmacological manipulation of mechanistic target of rapamycin complex 1 (mTORC1) further supported its involvement in edaravone-associated OPC differentiation and remyelination.

Together, these studies show that oligodendrocyte lineage maturation and myelin recovery can be pharmacologically enhanced after demyelination has developed in disease-relevant experimental systems. The delayed-treatment designs strengthen a remyelination interpretation, but neither study establishes human efficacy or the durability and functional integration of newly formed myelin.

Model context is critical. The clobetasol system was deliberately characterized by limited axonal injury and short follow-up, making it favorable relative to many human lesions. Edaravone has antioxidant, anti-inflammatory, neuroprotective and oligodendroglial effects, so preservation and reconstruction were not completely separated. Neither study establishes benefit in a necrotic lesion, an older host, a recurrently injured pathway or tissue under persistent antibody access.

### 4.5 Barrier 4: depletion of repairable substrate

Remyelination presupposes surviving axons. A distinct acute axonal pathology has been described in a spinal NMOSD model, indicating that axonal injury can develop early and cannot be reduced to late degeneration after myelin loss (28). In a longitudinal study of 51 first optic neuritis episodes, greater optic nerve lesion length predicted subsequent retinal neuroaxonal loss and visual outcome; only two participants had NMOSD, so the result is not an AQP4-specific validation study (29). Residual substrate is therefore a plausible upper bound on a remyelinating effect, not a validated eligibility threshold.

Remyelination as neuroprotection is most plausible for denuded but viable axons. Age, chronic denudation and greater axonal abnormality are expected to reduce responsiveness (9). Quantitative imaging does not yet solve the substrate problem: a small cross-sectional 7-tesla magnetic resonance imaging (7-T MRI) study detected group-level myelin-related signal differences in normal-appearing white matter but did not measure lesion repair or define a threshold (30). Preclinical studies should therefore model a range of substrate loss rather than selecting only lesions optimized to show remyelination.

### 4.6 Barrier 5: failure to transition from injury resolution to active repair

Resolution of overt inflammation does not necessarily create a reparative lesion environment. A lesion may show reduced complement activity and barrier closure while astrocytic support remains abnormal, debris accumulates, OPC differentiation stalls or axons continue to degenerate. Failure to enter active repair is therefore an integrated lesion state produced by different combinations of the other barriers, not a single pathway at the same biological level.

The five-barrier framework can incorporate these cross-cutting mechanisms without treating them as additional sequential stages. Microglia and macrophages, peripheral immune activity, mitochondria and other organelles, the neurovascular unit, and the extracellular matrix can modify several barriers simultaneously. **Figure 1** summarizes these interacting mechanisms without implying a fixed temporal sequence or uniform contribution across lesions.

**Figure 1:**
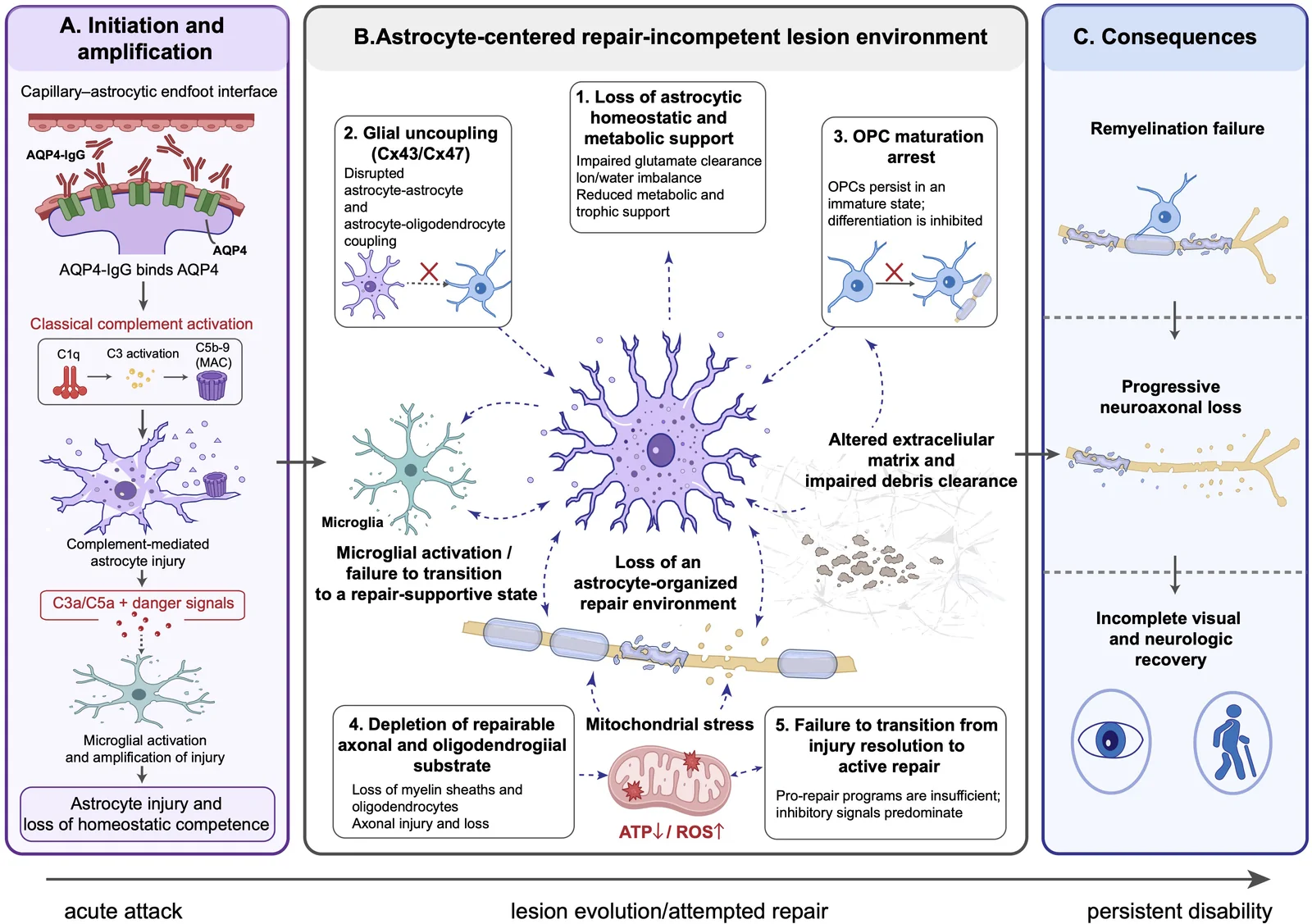
Candidate constraints on repair after AQP4-IgG-mediated injury. **(A)** Initiation and amplification: AQP4-IgG binding may cause sublytic astrocyte dysfunction or complement- and cell-amplified lytic astrocyte injury. **(B)** Astrocyte-centered repair-incompetent lesion environment: the schematic summarizes five linked constraints on repair: loss of astrocytic homeostatic support, glial uncoupling, impaired oligodendrocyte lineage progression, depletion of repairable neuroaxonal substrate, and failure to transition into an active reparative state. Complement activation and peripheral immune effector access contribute to ongoing injury, whereas microglial and macrophage debris and lipid handling, mitochondrial and other organelle stress, neurovascular dysfunction, and extracellular matrix remodeling may modify several repair domains simultaneously. **(C)** Consequences: remyelination failure, progressive neuroaxonal loss, and incomplete visual or neurological recovery.

## 5 Cross-cutting immune–glial determinants

### 5.1 Peripheral immune input and continuing effector injury

Repair occurs within the CNS, but peripheral immunity sets the conditions for renewed injury. Circulating AQP4-IgG alone does not indicate ongoing lesion activity; antibody access, barrier state, complement or Fc effector competence, and recruited granulocytes or monocytes must align. When they do, continuing astrocyte injury reduces the substrate available for repair and confounds interpretation of any downstream tissue signal (31).

The dominant mechanism changes with phase. Before an attack, B-cell and plasmablast activity, interleukin-6 signaling and circulating antibody set susceptibility without proving CNS injury. During an attack, classical complement activation, complement component 5a (C5a)-mediated recruitment, membrane attack complex-mediated injury and Fc-dependent neutrophil, eosinophil or monocyte effects can overlap. Early recovery begins as access and effector activity fall; stable remission may still include serum AQP4-IgG without continuing lesion exposure. Available evidence does not resolve the relative contributions of serum-derived and locally produced complement in human lesions.

Chronic immune evolution is plausible but incompletely defined. In one serial study, serum AQP4 recognition patterns varied over time in 7 of 20 participants (32). Intramolecular spreading is one interpretation; changes in polyclonal composition and assay variation are alternatives. No direct study identified in this review showed spreading from AQP4 to myelin or axonal antigens. Bystander inflammation and epitope spreading should therefore remain hypotheses, not explanations presented as established repair barriers.

### 5.2 Microglial and macrophage debris and lipid processing

Microglia have a larger role than lesion amplification alone. Astrocyte–microglia signaling contributes to evolving NMOSD lesions, while microglia and recruited macrophages can remove myelin and cellular debris, process lipid and generate signals associated with oligodendrocyte differentiation (33, 34). Persistent activation can be harmful, yet indiscriminate suppression can also be harmful if it prevents clearance of inhibitory debris.

NMOSD-relevant studies of triggering receptor expressed on myeloid cells 2 (TREM2) illustrate this duality. TREM2 deficiency impaired microglial accumulation, phagocytosis, degradation of myelin debris, oligodendrocyte proliferation and remyelination in an NMOSD model (35). In contrast, soluble TREM2 exposure was associated with phagocytic overloading, defective lysosomal and lipid metabolism, inflammatory persistence and impaired repair-related functions (36). Membrane receptor availability and soluble ectodomain signaling are mechanistically asymmetric and should not be interpreted as opposite positions on a single activation scale.

Common markers also do not cleanly distinguish resident microglia from recruited macrophages, and cerebrospinal fluid myeloid cells are not a direct readout of parenchymal microglia. The relevant construct is myeloid debris- and lipid-processing capacity: uptake relative to lesion load, lysosomal degradation and cholesterol export, considered alongside oxidative capacity and inflammatory output. Each component requires separate measurement and lineage attribution; total activation alone cannot guide development.

### 5.3 Organelle and metabolic stress

Organelle dysfunction and extracellular mitochondrial DNA signaling are candidate processes, not one undifferentiated “mitochondrial barrier.” In human induced pluripotent stem cell (iPSC)-derived astrocytes, NMO-IgG altered mitochondrial, endoplasmic reticulum and lysosomal organization and impaired bioenergetics and autophagy (37). A 2026 research report showed that small extracellular vesicles released by AQP4-IgG-stimulated astrocytes carried mitochondrial DNA and activated microglia through the toll-like receptor 9 (TLR9)–myeloid differentiation primary response 88 (MyD88)–NF-κB pathway; pathway inhibition attenuated the effect in cellular and mouse experiments (38). This supports an astrocyte-to-microglia injury-amplification route, not an independent post-injury repair barrier.

The repair implications operate at several levels. Astrocyte bioenergetic failure could impair glutamate and ion handling and reduce metabolic support to neighboring cells. Oligodendrocyte differentiation and synthesis of a large myelin membrane impose substantial biosynthetic and energetic demands. Demyelinated axons redistribute ion channels and require greater energy to sustain conduction. The first link has disease-relevant cellular support; the oligodendroglial and axonal links are supported mainly by broader glial and demyelination biology and should be labeled as cross-disease inference.

A metabolic intervention should specify the targeted cell, demonstrate that bioenergetic rescue precedes downstream benefit, and separate reduced inflammation from new myelin or axonal preservation. A change in mitochondrial morphology, respiration or oxidative stress alone is target engagement, not repair.

### 5.4 Neurovascular and extracellular matrix context

The extracellular matrix can affect OPC migration, differentiation and axonal contact, while the neurovascular unit—endothelium, pericytes, astrocytic end feet and perivascular basement membrane—determines antibody access and local recovery conditions. Direct AQP4-IgG-specific evidence remains limited. In multiple sclerosis tissue and generic demyelination models, high-molecular-weight hyaluronan accumulates in chronic lesions and inhibits OPC maturation (39). This is relevant indirect evidence, not proof that hyaluronan is a major barrier in AQP4-IgG-positive lesions. Indiscriminate matrix removal could also release bioactive fragments or destabilize the vascular barrier.

The first tractable study is spatial rather than therapeutic: map matrix composition and neurovascular compartments against astrocyte state, resident and recruited myeloid cells, OPC state and axonal integrity in human lesions. Disease-relevant models can then test a specified component after lesion burden has been prospectively matched. A matrix- or vascular-directed candidate should improve oligodendrocyte lineage progression and pathway function without worsening barrier integrity or acute inflammation.

### 5.5 Lesion site, age, timing and prior injury

Repair competence is unlikely to be a stable property of a patient. It is lesion- and intervention-specific. The same person may have an acutely salvageable optic nerve lesion, a previously necrotic spinal lesion and a sublytic brain lesion with very different capacities for astrocyte rescue, remyelination or axonal recovery. Age, time since injury, lesion volume, previous attacks and residual axonal population are expected to modify several barriers simultaneously.

These variables should be tested as continuous effect modifiers in models rather than converted prematurely into binary “repair-eligible” categories. A treatment that depends on OPC differentiation may fail when axonal substrate is depleted, whereas astrocyte rescue or acute neuroprotection may remain relevant earlier in the same lesion. **Figure 1** depicts interacting biological constraints whose relative importance is likely to vary with lesion state, site, and residual substrate. Representative evidence supporting the five barriers and cross-cutting determinants is summarized in **Table 2**.

**Table 2:** Representative evidence for the five barriers and cross-cutting determinants.

Domain	Study/model and provenance	Timing or endpoint	Supported inference	Principal limit
Human astrocyte states	Cross-sectional human pathology (12–14).	AQP4/GFAP, complement, morphology and transcript/protein expression.	Destructive, sublytic and repopulation-associated phenotypes exist.	The same lesion was not followed; morphology does not establish restored function.
Barrier closure and glial return	Focal AQP4-IgG/complement experimental lesions (15, 16).	Barrier closure, astrocyte/oligodendroglial return and myelin during recovery.	Barrier resealing, cell return and myelin measures can dissociate.	Human temporal sequence and causality were not established.
Longitudinal astrocyte repopulation	Focal cortical AQP4 antibody/complement ablation with human tissue comparison (18).	Repeated imaging, spatial transcriptomics, proliferation, multinucleation and nuclear translocation.	Defines how mature astrocytes can repopulate depleted territory.	Selective focal model; no proof of full homeostatic restoration or remyelination.
Astrocyte state	IFNAR1 and CHI3L1 experimental studies (22, 23).	Astrocyte activation, inflammatory signaling, demyelination and model severity.	Astrocyte state alters lesion outcome and is pharmacologically tractable.	Interventions overlap with evolving injury; post-injury repair was not isolated.
Glial coupling	NMOSD tissue/cultures, non-NMOSD connexin dependence and focal ablation model (18, 24, 25).	Expression, localization, transfer, hemichannel opening, stability and repopulation.	Repopulation and restoration of functional coupling are separable.	Physiological coupling in living human lesions and therapeutic direction remain unknown.
Organelle stress	Human iPSC-derived astrocytes exposed to NMO-IgG (37).	Organelle organization, bioenergetics and autophagy.	Astrocyte organelles and metabolism are perturbed.	Independent contribution to remyelination failure and benefit from metabolic rescue are unproven.
Extracellular vesicle mitochondrial DNA	AQP4-IgG-stimulated astrocyte vesicles in cellular and mouse experiments (38).	Vesicle mitochondrial DNA, TLR9/MyD88/NF-κB activation and microglial response.	Supports an astrocyte-to-microglia injury-amplification route.	Does not establish an independent post-injury repair barrier.
Myeloid debris and lipid processing	TREM2-deficient and soluble TREM2 NMOSD models (35, 36).	Debris uptake/degradation, lysosomal/lipid metabolism, oligodendrocyte response and myelin.	Myeloid processing can support or obstruct repair depending on mechanism and load.	Lineage attribution and a validated human proxy are lacking.
Matrix context	Multiple sclerosis tissue and generic demyelination models (39).	Hyaluronan and OPC maturation.	Matrix composition can restrict OPC maturation in other demyelinating disease.	Direct AQP4-IgG-specific evidence and therapeutic safety are unknown.
OPC maturation	Clobetasol and edaravone ex vivo/*in vivo* NMO-IgG/AQP4-IgG models (26, 27).	Post-demyelination dosing, mature oligodendrocytes, and myelin recovery; edaravone additionally included ultrastructure and a separate functional model.	Shows that myelin recovery can be pharmacologically enhanced after demyelination; edaravone provides stronger structural support for remyelination.	Favorable substrate, short follow-up, pleiotropy, incomplete functional integration, and no controlled human evidence.
Neuroaxonal substrate	Acute spinal AQP4-IgG/complement model and mixed-etiology optic neuritis cohort (28, 29).	Early axonal pathology and lesion length prediction of later retinal/visual loss.	Residual substrate plausibly limits a remyelinating effect.	No validated AQP4-specific eligibility threshold.
Myelin-related MRI signal	Cross-sectional 7-T study of 14 patients with NMOSD, 12 with multiple sclerosis and 10 controls (30).	T1 and magnetization-transfer-ratio histograms in normal-appearing white matter.	Detects group-level myelin-related abnormalities.	No lesion repair, longitudinal change or histological validation.

AQP4, aquaporin-4; CHI3L1, chitinase-3-like protein 1; GFAP, glial fibrillary acidic protein; IFNAR1, type I interferon receptor 1; iPSC, induced pluripotent stem cell; MRI, magnetic resonance imaging; MyD88, myeloid differentiation primary response 88; NF-κB, nuclear factor kappa B; NMO-IgG, patient-derived total immunoglobulin G from individuals with NMOSD; NMOSD, neuromyelitis optica spectrum disease; OPC, oligodendrocyte precursor cell; TLR9, toll-like receptor 9; TREM2, triggering receptor expressed on myeloid cells 2.

## 6 Mechanism-guided candidate strategies

**Figure 2** positions candidate interventions according to their principal biological role, recognizing that these therapeutic domains may overlap in time. The central distinction is whether an intervention prevents new injury, preserves substrate, restores a permissive function or forms new tissue. **Table 3** summarizes the present evidence and the next decisive experiment for each strategy.

**Figure 2:**
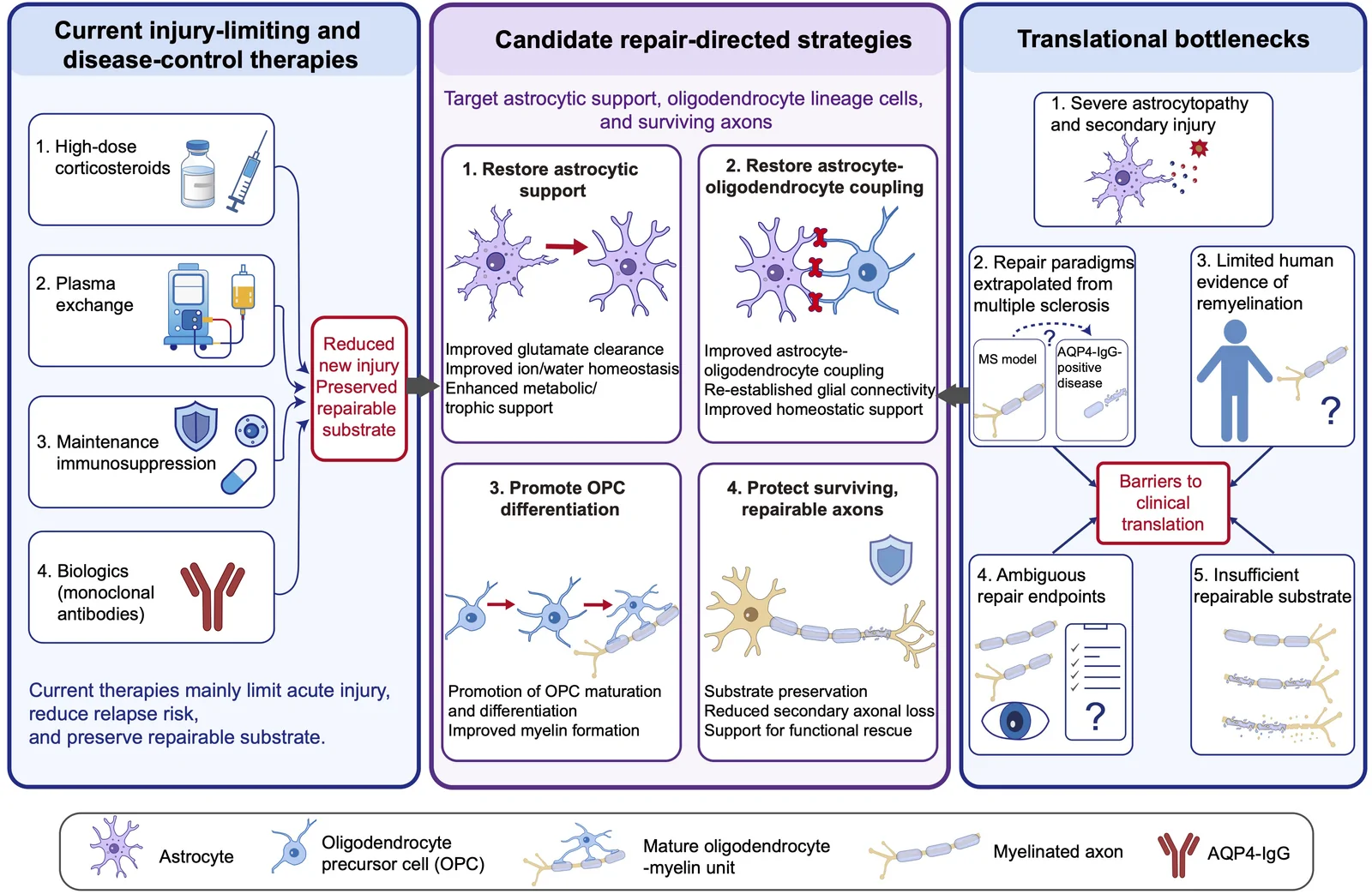
Therapeutic positioning after an AQP4-IgG-positive NMOSD attack. Therapeutic strategies are positioned according to their principal biological role. Disease control interventions reduce the risk of future attacks, whereas acute injury-limiting interventions aim to reduce ongoing tissue damage during an attack and preserve repairable substrate. Candidate strategies directed at astrocytes, the lesion microenvironment, or oligodendrocyte precursor cells (OPCs) aim to restore repair-permissive conditions or promote new myelin formation around surviving axons. These therapeutic domains may overlap in time. Major translational constraints include lesion heterogeneity, insufficient residual substrate, extrapolation from non-NMOSD models, drug delivery and safety, and outcome measures that cannot reliably distinguish tissue preservation, physiological recovery, and structural repair.

**Table 3:** Mechanism-guided candidate strategies and the next decisive experiment.

Strategy	Present evidence signal	Defensible current label	Next decisive experiment	Main failure mode
Maintenance complement, B-cell lineage and interleukin-6 receptor therapy	Controlled relapse prevention (4–6, 40).	Disease control and indirect substrate preservation.	Measure index lesion recovery prospectively under stable disease control.	Relapse prevention mislabeled as reconstruction.
Acute corticosteroids, plasma exchange and complement blockade	Observational human salvage evidence and repair-compatible preclinical recovery (41–43).	Acute injury limitation.	Burden-matched experiment or controlled add-on study that separates less initial injury from later repair.	Preservation explains the downstream signal.
Astrocyte regeneration or state modulation	FGF8–DBX1, focal repopulation, IFNAR1 and CHI3L1 models (17, 18, 22, 23).	Preclinical astrocyte replacement or reprogramming.	Astrocyte-specific necessity/rescue plus restored function and downstream myelin/physiology under persistent AQP4-IgG.	Cell return or reduced inflammation mistaken for functional repair.
Connexin-directed strategies	Disease-relevant coupling abnormalities with non-NMOSD mechanistic support (24, 25).	Mechanistically plausible, unvalidated repair-permissive approach.	Show correct localization and physiological transfer, then myelin and pathway benefit without hemichannel toxicity.	Direction-specific channel effects and no accessible human assay.
Myeloid debris and lipid processing strategies	TREM2-related NMOSD models (35, 36).	Preclinical repair-permissive approach.	Measure uptake relative to load, degradation, cholesterol export and downstream remyelination in lineage-resolved cells.	Global activation marker obscures productive versus overloaded states.
Organelle and metabolic modulation	Astrocyte organelle stress, vesicle mitochondrial DNA signaling and pleiotropic edaravone effects (27, 37, 38).	Target engagement or mixed injury-limiting/repair signal.	Cell-specific metabolic target engagement followed by reconstruction or axonal benefit under matched inflammation.	Nonspecific redox effects and mechanism ambiguity.
Clobetasol	Delayed post-demyelination increases in mature oligodendrocytes and myelin recovery in substrate-rich AQP4-IgG models (26).	Preclinical myelin repair signal.	Independent replication with lineage-labeled new myelin, ultrastructure, same-tract physiology, and exposure data.	Route, CNS exposure, glucocorticoid toxicity, short follow-up, and a favorable model.
Edaravone	Post-demyelination myelin recovery with ultrastructural and separate functional evidence; a registered AQP4-IgG-positive optic neuritis study (27, 44).	Preclinical remyelination evidence; human translation in progress.	Human safety, exposure, and mechanism-linked regional outcomes, with further testing of durability and same-pathway integration.	Pleiotropy, short follow-up, and no stand-alone human remyelination biomarker.
Remyelinating biologics	Opicinumab visual pathway trial outside AQP4-IgG-positive NMOSD (45).	Cross-disease design precedent.	Include disease-relevant exposure and target engagement assessment in early development.	Population mismatch and *post hoc* or per-protocol overinterpretation.
Stromal cells, extracellular vesicles and neurotrophic delivery	NMOSD animal data, an uncontrolled 31-participant phase 1/2a study and non-NMOSD exosome models (46–49).	Platform plausibility and early human safety/signal generation.	Product identity, biodistribution, cell-specific mediation, immune safety and controlled regional reconstruction outcome.	Manufacturing variability, mixed mechanisms and regression to the mean.

The evidence label refers to the strongest claim supported by the cited evidence, not to the eventual clinical potential of the strategy.

### 6.1 Disease control and acute injury limitation

Targeted maintenance therapies have established disease control in AQP4-IgG-positive NMOSD. Inebilizumab, eculizumab, satralizumab and ravulizumab reduce relapse risk in relevant populations (4–6, 40). Their indirect contribution to recovery is prevention of superimposed injury and preservation of axons, oligodendroglial cells and functional astrocytes. These trials were not designed to demonstrate repair of an established lesion.

High-dose corticosteroids and plasma exchange are classified as acute injury-limiting interventions because they are given while an attack is active. Earlier plasma exchange was associated with better outcome in a severe NMO spectrum cohort reported before contemporary serological classification, while a large optic neuritis series included AQP4-IgG-positive and seronegative NMOSD attacks without a randomized disease-specific timing estimate (41, 42). These observations support prompt salvage, not reconstruction.

Complement inhibition illustrates the same boundary. In AQP4-IgG-mediated experimental tissue, rapid C5 blockade stopped lesion extension and improved subsequent oligodendrocyte repopulation and myelin repair (43). Because blockade began while lesions were still extending, less initial injury can explain part or all of the downstream difference. The defensible label is acute injury limitation with repair-compatible recovery, not an isolated remyelinating effect.

### 6.2 Restoring astrocyte competence and glial coupling

Astrocyte-directed strategies can aim either to replace cells lost to lytic injury or to reprogram surviving or regenerated cells away from a maladaptive state. FGF8–DBX1 signaling supports the tractability of astrocyte regeneration; longitudinal focal ablation work defines a repopulation program; CHI3L1 pathway blockade and IFNAR1 manipulation show that astrocyte activation state can alter disease severity (17, 18, 22, 23). None of these studies proves that a full homeostatic program has been restored or that human tissue has been remyelinated.

Connexin-targeted treatment requires particular caution. Restoring physiological astrocyte–oligodendrocyte exchange may be beneficial, whereas indiscriminate opening or closing of hemichannels may worsen ionic imbalance or inflammatory signaling (24, 25). A candidate should demonstrate correct channel localization and intercellular transfer, then improved myelination and pathway physiology, without amplifying acute injury.

### 6.3 Correcting myeloid, metabolic and matrix dysfunction

TREM2-related experiments support manipulation of myeloid debris and lipid processing, but a therapeutic direction cannot be inferred from a single activation marker (35, 36). Similarly, the extracellular vesicle mitochondrial DNA study supports blockade of a harmful astrocyte-to-microglia signal, whereas edaravone and other metabolic candidates may affect several cell types and stages (27, 38). Matrix-directed strategies remain further upstream because the relevant molecule, molecular size, compartment and timing have not been established in AQP4-IgG-positive lesions (39).

For each of these strategies, an intervention should first change a prespecified function in the relevant cell or compartment. It should then improve a downstream construct—new myelin, axonal preservation or pathway physiology—after initial lesion burden and residual injury have been matched. A broad reduction in inflammatory staining is insufficient to identify a repair mechanism.

### 6.4 Direct remyelination and remyelinating biologics

Clobetasol and edaravone provide the strongest disease-relevant evidence that OPC maturation and myelin recovery can be enhanced after AQP4-IgG-mediated injury (26, 27). Further preclinical work should assess independent replication, CNS exposure, clinically realistic treatment delays, persistent antibody pressure, older or previously injured tissue, and whether newly formed myelin is structurally and physiologically competent. These gaps need not preclude a carefully monitored early human study when safety and exposure are acceptable, but they limit the strength of any remyelination claim.

Clinical translation of edaravone is being explored in a registered single-group study of AQP4-IgG-positive optic neuritis (44). The registration shows that human testing has begun; it does not establish efficacy or remyelination. Remyelinating biologics developed outside NMOSD can inform design but not efficacy assumptions. Opicinumab, an anti-LINGO-1 monoclonal antibody, did not meet the intention-to-treat primary visual evoked potential (VEP) endpoint in acute optic neuritis, although a per-protocol signal was reported (45). Its relevance here is the difficulty of exposure, timing and endpoint interpretation, not direct support for use in AQP4-IgG-positive NMOSD.

### 6.5 Cell, extracellular vesicle and neurotrophic approaches

Human umbilical-cord-derived mesenchymal stromal cells combine immunomodulatory and potentially trophic effects. An NMOSD animal study reported reduced inflammatory infiltration and myelin loss, preservation of astrocytes and neurons, and improved barrier integrity (46). A published 2026 single-arm phase 1/2a study in 31 patients reported tolerability and exploratory relapse-related, clinical, MRI and immunometabolic signals (47). Its uncontrolled before–after design cannot separate immunomodulation, natural history, regression to the mean and repair of an established lesion.

Mesenchymal stromal cell-derived exosomes and engineered exosomes carrying brain-derived neurotrophic factor (BDNF) promoted oligodendrocyte differentiation or remyelination in non-NMOSD demyelination models (48, 49). These studies support platform plausibility, not disease-specific efficacy. Extracellular vesicles can carry beneficial or harmful cargo; biodistribution, product identity, batch potency, cargo release, cell-specific target engagement and immune safety are prerequisites before an NMOSD repair claim is credible.

### 6.6 Overlapping treatment conditions and biological timing

A single-agent sequence may be unrealistic. Ongoing immune injury must be controlled, the local environment must support repair, and viable axons must remain when an OPC-directed treatment acts. These are overlapping biological conditions, not a fixed calendar sequence. An OPC-directed agent given during uncontrolled complement injury may generate cells that die or form unstable myelin; the same agent given after major axonal loss may be biologically active but clinically silent.

Timing should therefore be anchored to mechanism and directly observed lesion context. Recent lesions provide more substrate but more continuing injury and spontaneous recovery. Stable residual lesions offer cleaner attribution but less substrate. Preclinical work should test several biologically defined windows rather than select one favorable time point and label it “the repair phase”.

## 7 Demonstrating repair without confusing it with salvage or recovery

### 7.1 Minimum preclinical evidence for biological repair

The main preclinical error is to infer remyelination from more myelin protein, more oligodendrocytes or better behavior without establishing when demyelination occurred and what tissue was preserved. A biological remyelination experiment should document prior demyelination and surviving axons before treatment, prospectively match initial lesion burden, and start the intervention after the injury relevant to the claim has been established. Terminal histology can confirm group equivalence but cannot create a baseline match *post hoc*.

Newly differentiated oligodendrocytes or newly formed myelin should be identified by lineage tracing, pulse labeling or an equivalent method. Electron microscopy should test compact myelin and axon–myelin apposition; g-ratio analysis should be interpreted with axon caliber and sampling strategy; nodal and paranodal organization should be assessed; and physiology should be obtained from the same tract or pathway. These readouts address different failure modes and are not interchangeable.

Mechanistic attribution requires more than temporal association. Astrocyte-directed benefit should be lost with astrocyte-specific interruption of the proposed pathway or restored by a cell-specific rescue. Myeloid-directed benefit should link uptake relative to debris load, lysosomal degradation and lipid export to downstream myelin. Metabolic rescue should precede lineage or axonal benefit in the targeted compartment. Factorial or delayed-treatment designs are preferable when the candidate also reduces acute inflammation.

Durability and disease pressure matter. Follow-up should extend beyond the period of transient marker expression, and the repaired tissue should be tested during persistent low-level AQP4-IgG exposure or re-challenge. A treatment that works only after antibody and complement are removed may still be useful, but the claim must be limited to that context. Randomization, blinding, prespecified exclusions, biological replication and independent replication remain basic requirements rather than optional refinements.

### 7.2 A minimal bridge to human repair claims

No single human measure identified in this review proves remyelination. Conventional MRI defines lesion location, extent, enhancement, cavitation and gross tissue loss; quantitative MRI can provide myelin-related signals, but edema, gliosis, axonal density and partial-volume effects remain important confounders (30). In the visual pathway, optical coherence tomography (OCT) measures retinal neuroaxonal structure rather than optic nerve myelin. Acute peripapillary retinal nerve fiber layer edema and chronic floor effects limit interpretation, and primary retinal AQP4-IgG-related injury is biologically possible (50).

Pattern-reversal VEPs provide conduction information. Delayed peak time is compatible with demyelination, while reduced amplitude can reflect axonal loss, retinal dysfunction, desynchronization, acuity or fixation (51, 52). A shorter P100 peak time after treatment is compatible with improved conduction but can also reflect edema resolution, temperature, attention or resynchronization. The current International Society for Clinical Electrophysiology of Vision standard uses “peak time” rather than “latency” for this measurement (53).

Spinal cord and brainstem outcomes must be region-specific. Improvement in motor, sensory, sphincter, pain, swallowing, ocular motor, or area postrema manifestations is clinically meaningful but may reflect reduced inflammation, physiological recovery or compensation; area postrema involvement is a core NMOSD presentation (54). Serum GFAP and neurofilament light chain (NfL) provide whole-CNS context for astrocytic and neuroaxonal injury, not regional reconstruction (55, 56).

A clinically meaningful human repair claim therefore requires longitudinal convergence across different constructs: documented prior injury and residual substrate; intervention-linked structural or cellular restoration; an orthogonal physiological or regional functional benefit; and durability while recurrent attacks, treatment changes and ongoing injury are quantified. A human remyelination claim additionally requires myelin-sensitive evidence linked to preserved axons and improved conduction in the same pathway. **Figure 3** summarizes the minimum evidence domains needed to support a human repair or remyelination claim.

**Figure 3:**
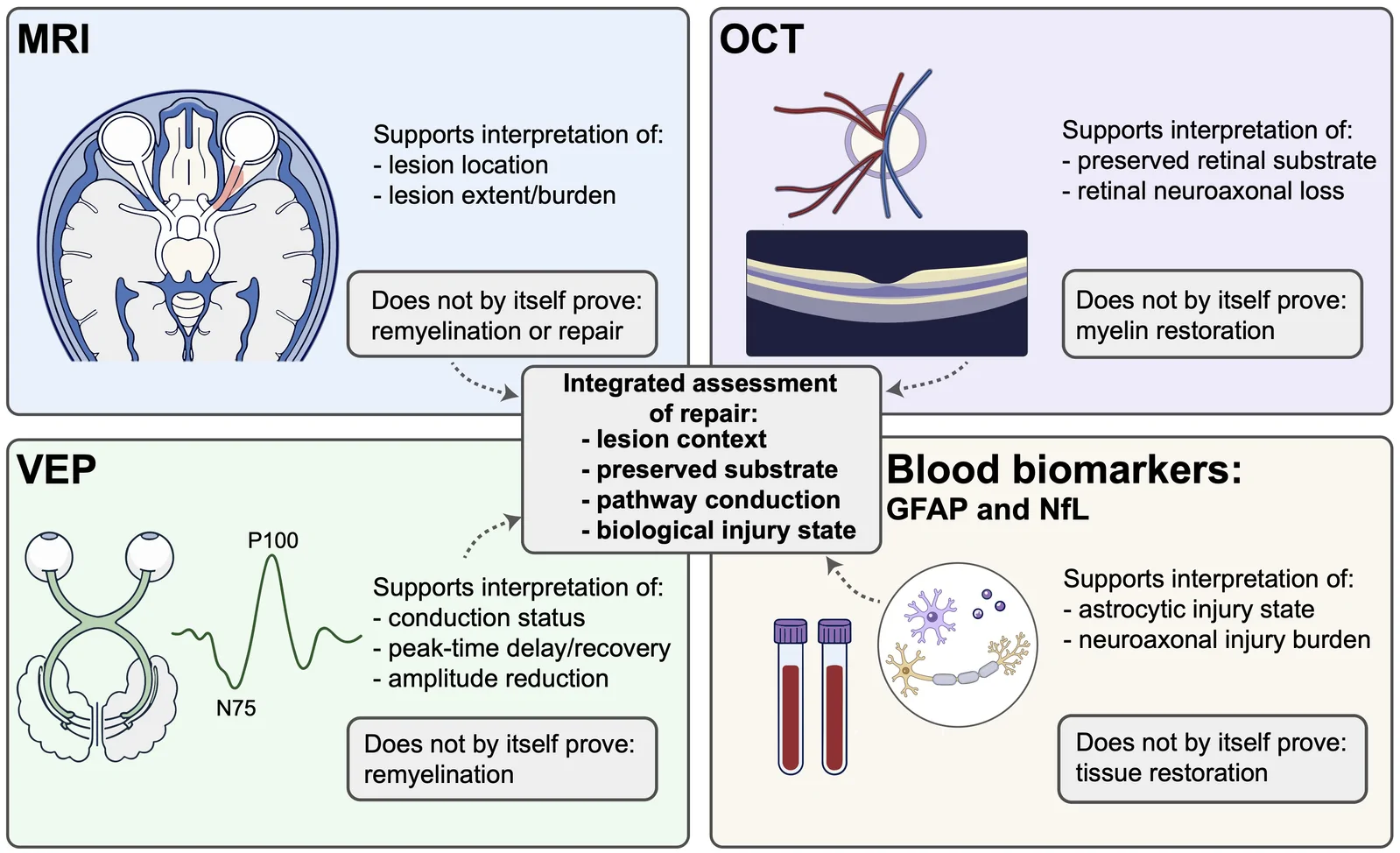
Multimodal assessment of optic pathway repair. Orbital magnetic resonance imaging (MRI) defines optic nerve lesion context and structural burden; optical coherence tomography (OCT) quantifies retinal neuroaxonal substrate and loss; pattern-reversal visual evoked potential (VEP) peak time and amplitude provide measures of visual pathway physiology; and serum glial fibrillary acidic protein (GFAP) and neurofilament light chain (NfL) provide a global context for astrocytic and neuroaxonal injury across the central nervous system. Each measure has important limitations, including edema-related MRI changes, OCT segmentation and floor effects, unrecordable VEPs, concomitant retinal pathology, and the non-regional nature of circulating biomarkers. No single measure establishes remyelination; interpretation therefore requires longitudinal alignment across structural, physiological, and clinical outcomes, together with a durable regional patient-relevant benefit.

## 8 Decisive experimental priorities

### 8.1 Map human lesion heterogeneity at cellular and spatial resolution

One priority is a donor-replicated, anatomically annotated spatial atlas of AQP4-IgG-positive lesions. Astrocyte polarity and homeostatic proteins, connexins, resident and recruited myeloid cells, debris and lipid load, OPC state, mature oligodendrocytes, myelin, axonal integrity, matrix and neurovascular compartments should be measured in the same tissue. Donor-level or hierarchical analysis is essential; thousands of cells from one donor do not substitute for biological replication. Cross-sectional tissue can identify coexisting states but cannot establish temporal order.

### 8.2 Separate cell return from functional restoration

Longitudinal models should track astrocyte repopulation and then ask whether returning cells recover AQP4 polarity, EAAT2 and Kir4.1 function, physiological gap junction transfer, metabolic support and neurovascular integration. Oligodendrocyte maturation, new myelin, nodal organization, conduction and durable function should be measured downstream. The critical comparison is not more GFAP-positive cells versus fewer cells; it is functional restoration with and without cell-specific interruption of the proposed mechanism.

### 8.3 Distinguish productive myeloid processing from inflammatory overload

Myeloid studies should quantify debris uptake relative to load, lysosomal degradation, cholesterol export, oxidative state and inflammatory output in lineage-resolved resident microglia and recruited macrophages. TREM2 abundance or shedding can be included, but neither should be used as a stand-alone dose target. A useful perturbation must improve downstream oligodendrocyte progression or myelin under matched initial lesion burden rather than merely lower ionized calcium-binding adapter molecule 1 staining.

### 8.4 Prove new myelin rather than preserved myelin

Delayed-treatment experiments should use a non-terminal baseline measure or a prospectively validated stratification proxy to match demyelination and axonal substrate before randomization. Lineage tracing, pulse labeling, electron microscopy, g-ratio, axon–myelin apposition, nodal and paranodal organization, and same-pathway physiology should be integrated. Preservation-only and repair-directed comparator arms can help identify whether a candidate works by reducing initial injury, forming new tissue or both.

### 8.5 Test repair under persistent disease pressure and across biological diversity

Models that remove AQP4-IgG and complement before treatment create an unusually permissive recovery phase. Candidates should also be tested during persistent low-level antibody exposure, renewed barrier access or re-challenge. Age, sex, lesion site, prior injury, recurrent attacks and the degree of axonal loss should be treated as planned sources of heterogeneity. A finding confined to young animals with a focal substrate-rich cerebral lesion is proof of principle, not a general NMOSD repair program.

### 8.6 Use proportionate criteria for early human translation

Human testing need not wait until every mechanistic uncertainty has been resolved. When a candidate has a credible biological rationale, acceptable nonclinical safety, a feasible route and a plausible CNS exposure strategy, a small early-phase study can assess safety, pharmacokinetics, pharmacodynamics and exploratory regional outcomes. The evidentiary requirement should be proportional to product risk and prior human experience: a repurposed drug with an established safety record may reasonably enter exploratory testing earlier than a novel biologic, cell product or gene therapy. This flexibility concerns whether a study may begin, not how strongly its results may be interpreted.

**Table 4** summarizes experimental designs that would strengthen mechanistic interpretation and confidence in a repair signal. A candidate should not be described as remyelinating on the basis of one favorable cell count, myelin protein stain, or global functional score, but incomplete mechanistic evidence does not automatically preclude a carefully monitored safety or exploratory study.

**Table 4:** Decisive experiments for a preclinical NMOSD repair program.

Question	Study design	Primary mechanistic readout	Orthogonal downstream readout	Decision rule
Which lesion states coexist in human tissue?	Donor-replicated, anatomically annotated spatial pathology with hierarchical analysis.	Astrocyte polarity/function, resident and recruited myeloid cells, matrix, neurovascular, OPC and axonal states.	Replication across donors and a hypothesis transferable to a longitudinal model.	Cross-sectional tissue cannot establish temporal order; cell-level associations from a single donor require biological replication.
Does astrocyte return restore function?	Longitudinal focal model with lineage tracing and astrocyte-specific perturbation or rescue.	AQP4 polarity, EAAT2/Kir4.1 function, gap junction transfer, metabolic and neurovascular support.	OPC maturation, new myelin, nodal organization, conduction and durability.	No repair claim from GFAP-positive cell number or barrier closure alone.
Is a myeloid response productive or overloaded?	Lineage-resolved perturbation with matched debris/lipid load and initial lesion burden.	Uptake relative to load, lysosomal degradation, cholesterol export, oxidative and inflammatory output.	Oligodendrocyte progression, myelin and pathway physiology.	Total activation, Iba1, or soluble TREM2 alone cannot guide dosing or advancement.
Is the signal new myelin or preserved myelin?	Randomized, blinded delayed-treatment experiment with a non-terminal baseline measure or validated stratification proxy.	Lineage- or pulse-labeled new oligodendrocytes/myelin and ultrastructure.	Axon–myelin apposition, g-ratio, nodal/paranodal structure and same-pathway physiology.	If benefit disappears after initial injury is balanced, interpret it as injury limitation; myelin protein change alone is insufficient to establish new myelin.
Does repair survive continued disease pressure?	Persistent low-level AQP4-IgG exposure, renewed barrier access or re-challenge after apparent repair.	Stability of the restored cellular function or new myelin.	Axonal survival, conduction and function after re-challenge.	A context-limited claim is required if benefit depends on complete antibody/complement withdrawal.
Is the effect generalizable?	Replication across age, sex, lesion site, prior injury, recurrent attacks and substrate loss severity.	Consistency of target engagement and direction of effect.	Durable structural and physiological benefit across prespecified strata.	A young, focal, substrate-rich model is proof of principle, not a general repair program.
Is human translation pharmacologically plausible?	Human-relevant exposure and pharmacodynamic study and, where feasible, a small prospective treated recovery cohort.	CNS exposure and mechanism-linked pharmacodynamic signal.	Reliable longitudinal regional structure, physiology and function.	Early human study may be reasonable when safety, route, and exposure strategy are acceptable; the strength of efficacy and repair claims should reflect assay reliability and compatibility with standard treatment.

These considerations are qualitative and intended to guide interpretation; universal numerical thresholds have not been validated for NMOSD repair studies.

## 9 Discussion

AQP4-IgG-positive NMOSD is difficult to repair because the lesion does not simply lose myelin. It loses the astrocyte-organized environment that coordinates ion and glutamate homeostasis, metabolic support, glial communication, immune containment, and neurovascular function. The five linked barriers remain a useful top-level model: loss of astrocytic support, glial uncoupling, OPC maturation stall, depletion of repairable substrate, and failure to enter an active repair program. Peripheral immune input, myeloid processing, organelle stress, and matrix or vascular context can modify several barriers at once.

The evidence is uneven. Human pathology establishes heterogeneity but not the history of one lesion. Longitudinal focal ablation work demonstrates substantial astrocytic plasticity and a mechanism of territory repopulation, but does not show that a destructive human lesion follows the same trajectory or that returning cells regain all homeostatic functions. Disease-relevant models allow causal perturbation, yet many use focal, substrate-rich injury, start treatment while injury is evolving or remove antibody and complement before the repair phase. These design features can overestimate the tractability of human lesions.

The distinction between biological and clinical claims is therefore essential. Delayed-treatment studies of clobetasol and edaravone show that oligodendrocyte maturation and myelin recovery can be enhanced after demyelination has developed; edaravone adds ultrastructural evidence compatible with remyelination and functional improvement in a separate systemic model (26, 27). These remain preclinical signals. Maintenance immunotherapies establish relapse prevention, whereas acute complement blockade and plasma exchange primarily limit injury. Two uncontrolled 2026 case series used C5 inhibitors as add-on rescue in severe AQP4-IgG-positive attacks (57, 58). These observations may justify controlled studies of acute injury limitation, but selection, prior rescue, treatment timing, and regression to the mean preclude a repair claim.

Most confirmed disability accumulation in an international AQP4-IgG-positive cohort was relapse-associated, and progression independent of relapse activity was uncommon (59). Retinal thinning has nevertheless been reported in AQP4-IgG-positive eyes without recognized optic neuritis (60). Subclinical or attack-independent injury is therefore possible, but poor recovery or a biomarker change does not prove persistent inflammation in an index lesion. Repair studies should measure continuing injury rather than infer it from outcome.

The article also has limitations. The search was narrative rather than systematic, newly published or unindexed work may have been missed, and several important domains rely on cross-disease evidence. “Repair competence” remains a latent biological construct, not a validated clinical category. The five barriers are organizing devices rather than equally weighted, sequential stages. Their value lies in generating discriminating experiments and preventing category errors—not in claiming that every lesion follows one pathway.

Major scientific opportunities remain preclinical. Human spatial pathology can identify which cellular states coexist; longitudinal and delayed-treatment experiments can test causality; persistent-antibody and re-challenge models can assess durability; and lineage-resolved ultrastructure plus same-pathway physiology can distinguish new myelin from preservation. Early human studies may proceed in parallel when safety, route, and exposure are acceptable, particularly for repurposed agents with prior clinical experience, while interpretation remains proportional to the evidence.

## 10 Conclusions

Cell return, barrier closure, reduced inflammation, or clinical improvement after an AQP4-IgG-positive NMOSD attack cannot be assumed to represent tissue repair. The five linked barriers remain useful, with peripheral immune activity, myeloid debris and lipid processing, organelle stress, and neurovascular or matrix context acting as cross-cutting determinants. Disease-relevant preclinical studies show that oligodendrocyte maturation and myelin recovery can be enhanced after established demyelination, but no controlled human study has demonstrated remyelination of an established AQP4-IgG-positive NMOSD lesion.

Further progress will come from experiments that confirm established injury, distinguish preservation from newly formed myelin, and test durability under persistent disease pressure. These studies can proceed alongside proportionate early-phase human evaluation when safety, exposure, and biological rationale are adequate. Human findings should be interpreted according to the construct actually measured, with clinical improvement, reduced injury, and remyelination kept distinct.
